# Editorial: Insights in pediatric cardiology: 2021

**DOI:** 10.3389/fped.2022.1059894

**Published:** 2022-12-05

**Authors:** Ruth Heying, Antonio F. Corno

**Affiliations:** ^1^ Department of Pediatric Cardiology, University Hospitals Leuven, Leuven, Belgium; ^2^ Children Heart Institute, Children’s Memorial Hermann Hospital, UT Health Science Center, McGovern Medical School, Houston, TX, United States

**Keywords:** pediatric cardiology, insights, imaging, kawasaki disease, fontan, future perspectives

**Editorial on the Research Topic** Insights in pediatric cardiology: 2021

The rapid introduction of innovations in the clinical practice of pediatric cardiology, covering the entire field from the basic sciences to bedside application, has prompted us to promote this Research Topic on “Insights in Pediatric Cardiology: 2021”.

This editorial initiative focuses on new insights, novel developments, current challenges, and future perspectives in the field of pediatric cardiology. Associated members of accomplished editorial boards have contributed with various topics, shedding light on current challenges, inspiring and providing direction to researchers in the field. Major progress has been made, especially in genetics, and various imaging techniques have been developed, allowing improvement in diagnoses and therapeutic options. Inflammatory diseases and associated general health care factors have gained increasing interest in the last decade.

We have collected 16 articles over the past 18 months in this Research Topic, and are pleased to introduce them to readers of *Frontiers in Pediatrics*.

## Imaging modalities

One sector of pediatric cardiology where the most striking progress has been made is imaging. The advent of three-dimensional (3D) and four-dimensional (4D) reconstructions of the heart, as well as systemic and pulmonary circulations, has completely changed the knowledge on all details required for decision-making processes. Conventional images were the only images available a few years ago and are nowadays not sufficient in patient presentation. In this regard, Bui et al. (2021) presented the advantages of diagnostic modalities involving 3D visualization, such as 3D printed anatomical models as well as virtual and augmented reality; not only advantageous for clinical practice, but also for teaching and training. The review article analyzed the use of these new technologies in medical education, and included a thorough and honest report of their benefits and limitations.

An interesting study was reported by Ardakani et al. (2022) who studied, in aortic coarctation, the relationship between aortic shape and hemodynamic parameters by means of computational simulations, purposely isolating morphological variables. Using aortic geometries derived from magnetic resonance imaging, computational simulations using a statistical shape modeling methodology were run in three aortic models: the absence of, surgically repaired, and unrepaired aortic coarctation. Even small alterations in the aortic morphology had an impact on key hemodynamic indices. The effective management of aortic coarctation affects long-term cardiovascular outcome phenomena, such as persistent hypertension in the absence of any clinically significant narrowing; this may be explained by differences observed in the aortic morphology.

In another retrospective study on aortic coarctation, Tuo et al. (2022) investigated the fetal echocardiographic parameters in the second and/or third trimester of pregnancy for all suspected cases aortic coarctation, in order to improve prenatal prediction after birth. Neonates were divided into two groups depending on the presence or absence of aortic coarctation after birth, and the criteria used for fetal diagnosis of aortic coarctation allowed accurate diagnosis of the most severe cases; however, the rate of false positives was relatively high for milder cases.

Closure of patent ductus arteriosus using interventional catheter techniques is still performed under fluoroscopy visualization. Many years after the first successful interventional closure under transesophageal echocardiographic guidance, Siagian et al. (2022) investigated the feasibility and safety of patent ductus arteriosus closure under echocardiography-only guidance over 1 year, comparing fluoroscopy and echocardiography-guided procedures in two groups of 30 children each. Primary endpoint procedural success was recorded, as well as secondary endpoints procedural time and the rate of adverse events. All patients presented successful closure of the patent ductus arteriosus, with residual shunts disappearing during follow-up in both groups. The procedural time was not significantly different between the fluoroscopy and echocardiography group.

## Kawasaki disease

Because of migration in the last few decades, typical diseases like Kawasaki disease are now observed more frequently worldwide. Since the factors predicting high-risk Kawasaki disease remain unclear, Liu et al. (2022) studied the risk factors for resistance to intravenous immunoglobulin treatment and coronary artery aneurysm development in a high-risk Chinese pediatric population, comparing the performances of 11 scoring systems in a population of 346 children between 2013 and 2021. From the Kobayashi score and five Chinese system scores (Tang, Yang, Lan, Liping, and Wu), results of curve analyses were observed to be highest in the Liping scoring system for intravenous immunoglobulin resistance. The researchers concluded that the Liping scoring system is the most appropriate method to identify high-risk patients with Kawasaki disease for intravenous immunoglobulin resistance in the Chinese pediatric population.

Resistance to intravenous immunoglobulin therapy was also addressed by Li et al. (2022) The authors investigated the best available scoring models for the early identification of intravenous immunoglobulin-resistant diseases. In a very large patient population of >1,000 children across two separate hospitals, 15 variables in the training set were statistically different between intravenous immunoglobulin-sensitive and -resistant, including rash, duration of fever, peripheral blood neutrophil-to-lymphocyte ratio, prognostic nutritional index, percentage of monocytes and eosinophils, as well as high-sensitivity C-reactive protein. Based on the results of this study, a scoring model with elevated sensitivity, specificity, and accuracy of prediction was constructed to predict intravenous immunoglobulin resistance, which can greatly assist pediatricians in developing a suitable therapeutic strategy for these children.

In another interesting study on Kawasaki disease, Van Stijn et al. (2022) conducted an extensive literature review to identify patients with Kawasaki disease and their potential to develop coronary artery pathology, particularly coronary artery aneurysms, using an integrated cardiovascular program tailored to the severity of the existing coronary artery pathology. Their practical workflow included expert opinions from pediatric cardiologists, infectious disease specialists, and radiology experts, who should be able to anticipate the risk of coronary artery aneurysms in patients with Kawasaki disease.

## Fontan circulation

Although the Fontan procedure has dramatically improved survival rates in patients with a single ventricle, long-term outcomes are still complicated by significant morbidity, with complications mainly due to elevated systemic venous pressure, which is characteristic of Fontan circulation. Consequently, these patients need close clinical and imaging monitoring, since early recognition of failing hemodynamics can be essential for long-term survival. Although echocardiography remains the first-in-line monitoring modality method, nowadays, cross-sectional imaging has become an essential tool in the evaluation of these patients. Ciliberti et al. (2022) analyzed the role of other diagnostic techniques, underlining the importance of cardiac magnetic resonance, particularly in simultaneously providing anatomic and functional information as well as essential information about extra-cardiac complications such as liver dysfunction and/or abnormal lymphatic drainage. Cardiac computerized tomography scanning may be useful when magnetic resonance is contraindicated, or for anatomical assessment in uncooperative patients. Cardiac catheterization is currently reserved to measure specific hemodynamic parameters and for interventional procedures.

While the early advantages of fenestration in the Fontan procedure are well known, little evidence exists regarding the long-term consequences of Fontan fenestration and the effects of interventional closure of the fenestration when induced by the clinical situation. Because of this, Greenleaf et al. (2022) performed a systematic literature review and a meta-analysis of the latest outcomes of Fontan fenestration, analyzing 922 publications for parameters such as changes in oxygen saturation, cavo-pulmonary pressure, maximum heart rate during exercise, exercise duration, and oxygen saturation after fenestration closure. In 877 patients that received interventional catheter closure of the fenestration, at a mean interval of 27.4 months after fenestration closure, a significant increase in arterial oxygen saturation and exercise duration was determined; however, an associated increase in the mean cavo-pulmonary pressure also occurred. This study concluded that late closure of a Fontan fenestration improves resting oxygen saturation, exercise oxygen saturation, and exercise duration. Concrete conclusions remain guarded in relation to the management, creation, or enlargement of Fontan fenestrations. The lack of comparative data suggests the need for improved prospective trials to examine the role of fenestration in the management of late Fontan failure.

## Associated health care issues

One of the most frequent health issues affecting current society is obesity, with significant consequences on the mortality and morbidity of adult populations, requiring a large number of resources and efforts by caregivers and society at large. Lee et al. (2021) screened a population of overweight adolescents with metabolic syndrome, and obtained a risk stratification for future cardiovascular disease in this population. Undoubtedly, this study will stimulate further investigations on the early development of obesity and the associated co-morbidities.

Children with congenital heart defects have a well-established risk of neuropsychiatric co-morbidities, including attention deficit/hyperactivity disorder (ADHD) symptoms; it is unknown whether the higher burden of ADHD symptoms is mainly driven by hyperactivity, inattention, or both. Lau-Jensen et al. (2021) compared a group of patients with simple congenital heart defects with age-matched controls and found a higher symptom burden across all ADHD scores and all symptom sub-scores, which was driven by both inattention and hyperactivity symptoms; inattention symptoms were more prominent. The authors suggested routine screening for ADHD symptoms in all children with congenital heart defects to facilitate adequate help and guidance since these symptoms are easily overlooked.

One of the leading causes of sudden, unexplained death of infants is sudden infant death syndrome (SIDS). While this dramatic health issue has been progressively recognized over the last few years, we are still far from an adequate policy for its prevention. Yamada et al. (2021) reported a research project to identify associations between SIDS and race/ethnicity, birth weight/gestational age, and socioeconomic/environmental factors in the referral area of North Carolina, USA. Based on the results of the study, since maternal, socioeconomic, and environmental risk factors are all associated with a higher incidence of SIDS in the population investigated, a thorough risk assessment should be performed in all infants at risk.

Returning to the field of connective tissue disease affecting systemic circulation, neurofibromatosis, also known as von Recklinghausen disease, a common autosomal dominant disorder caused by mutations in the *NF1* gene, is associated with early-onset systemic hypertension. Lu et al. (2021), in addition to a systematic literature review, performed a study on *NF1* mutations in two unrelated Chinese families with NF-1, who presented with early-onset hypertension. Using whole-exome sequencing, the authors identified one recurrent and one novel frame-shift mutation. Because hypertension is not a rare complication of *NF-1*, considering the phenotypic heterogeneity of *NF-1*, and based on the observational results, the authors suggested genetic testing as a tool for early and accurate diagnosis, especially in children and adolescents, to potentially prevent serious cardiovascular events.

## Connective tissue disease

Connective diseases affecting the aorta, such as Marfan and Loeys-Dietz syndrome, have attracted particular interest due to the progress achieved in the accurate diagnosis of cardiovascular malformation using 3D imaging and the subsequent improvement in surgical outcomes. This approach has also allowed the treatment of an increasing number of adolescents and children. Camarda et al. (2022) investigated pediatric patients with Marfan and Loeys-Dietz syndrome and other undifferentiated connective tissue diseases, using magnetic resonance imaging to determine patient-specific fluid structure interaction models. The time-averaged wall shear stress and oscillatory shear index were measured and compared with age- and gender-matched control participants. The authors reported that differences in these two parameters were apparently driven by local morphological differences, particularly the dimensions of the ascending aorta and the cardiac output. This is another example of a potential foundation for larger future studies on connective tissue disorders in the pediatric population.

## Right ventricular hypoplasia

Hirono et al. (2022) performed a systematic literature review of isolated right ventricular hypoplasia as a rare congenital myocardial disease, not associated with severe pulmonary or tricuspid valve malformation, and focused on evaluating clinical statuses and outcomes. The result of the study was that an early diagnosis of isolated right ventricular hypoplasia in children with cyanosis is associated with a high mortality.

## Postural tachycardia

Postural tachycardia syndrome in children is quite a rare disease, but with serious potential consequences. Wang et al. (2022) investigated the predictive values of heart rate and blood pressure on the prognosis of postural tachycardia syndrome in a large group of children. The predictive value of the four combined indicators for prognosis was superior to that of the single factor, conventionally used. Based on these observations, the authors divided the patients into two groups: good and poor prognosis, and treated them accordingly, with encouraging outcomes.

## Conclusion

The various interesting contributions collected in this Research Topic highlight key matters on the pathophysiology of congenital heart disease and its diagnostic management, herewith indicating future directions to improve the treatment of patients.

